# New horizons of microphysiological systems: India forging its path in human-relevant research

**DOI:** 10.1242/bio.059847

**Published:** 2023-04-18

**Authors:** Surat Parvatam, Kasturi Mahadik, Anushka Banerjee, Kadambari Patil, V. Radha, Madhusudhana Rao

**Affiliations:** ^1^ Centre for Predictive Human Model Systems, Atal Incubation Centre-CCMB, Hyderabad 500039, India; ^2^ CSIR-Centre for Cellular and Molecular Biology, Hyderabad 500007, India

**Keywords:** Microphysiological systems, Organ-on-chip, Organoid, Stem cell biology

## Abstract

The past decade has seen expeditious developments in our ability to grow and maintain a variety of human cells and tissues, with properties closely mimicking those in the human body. Prominent researchers and entrepreneurs from all over the world assembled in Hyderabad, India to discuss developments in this field that have not only aided fundamental understanding of organ development and disease processes but have served as good physiological models for toxicity testing and drug development. The speakers presented ingenious, cutting-edge technology and forward-thinking ideas. This report presents the salient aspects of their discussions, highlights the importance of identifying unmet needs, and discusses setting of standards that will help regulatory approvals as we move into a new era, with nominal animal use in research and effective drug discovery.

## Teaser

**Summary:** Parvatam and colleagues elaborate on India's first meeting focused on Microphysiological Systems and how they hope to usher in a new era of 3D biology in the country.

## Introduction

The use of microphysiological systems (MPS) including organoids and organs-on-chip has been steadily increasing globally in areas of basic biomedical research and drug discovery (Marx et al., 2016, 2020; Roth and MPS-WS BERLIN, 2019, 2021). This rise has been attributed to a growing realisation that animal models are often limited in their ability to mimic human biology (Seok et al., 2013). Human organoids, derived from stem cells, have now enabled recapitulation of structure and function of human organs to a great degree (Kim et al., 2020). Also, patient-specific stem cell derived organoids serve as out-standing *in vitro* developmental models to study various human diseases and for evaluating novel therapeutic drugs. Similarly, microfluidic devices lined with human cells, termed as organ-on-chip (OoC), mimic various aspects of organ-level physiology and pathology (Ingber, 2022).

There has been a rise in the adoption of these technologies globally. According to the recent legislation signed by the US President, new medicines do not need to be tested on animals to receive the US Food and Drug Administration approval (Wadman, 2023). This landmark decision allows the incorporation of non-animal methodologies to study the safety and efficacy of drugs. In addition, several countries, including the US, Europe, China, Japan, and Canada have set up government-funded Alternatives to Animal Research Centres towards development and validation of these new human-relevant methods.

While the field of MPS is still naïve in India, in the past five years, more than 50 research labs and companies have begun working on MPS in India (Parvatam et al., 2020). Centre for Predictive Human Model Systems (CPHMS) at the Atal Incubation Centre - Centre for Cellular and Molecular Biology (AIC-CCMB), jointly established by AIC-CCMB and Humane Society International (HSI)-India, is a science and policy centre to enable scientific advances using human-relevant systems in India. These include MPS, advanced human imaging and computational *in silico* tools, among others, that are based on human, rather than animal biology. This is done via a multi-pronged approach that includes education and training activities, raising public awareness, policy research, enabling stakeholder crosstalk, liaising with regulatory bodies and funding research activities. Despite the growing interest in this field, there have been very few scientific meetings or training opportunities to bring together this community in India.

Towards this aim, CPHMS and the Council of Scientific and Industrial Research-Centre for Cellular and Molecular Biology (CSIR-CCMB) recently conducted a five-day India | EMBO lecture course on Microphysiological Systems from Oct 31-Nov 4, 2022, in Hyderabad, India. This was India's first meeting with a collective focus on organoid and OoC research. It brought together the growing community of academic and industry MPS researchers currently working *in silos*. In this meeting report, we highlight the key takeaways from the meeting and a vision for the future.

## Speaker and participant distributions

**Fig. 1.**
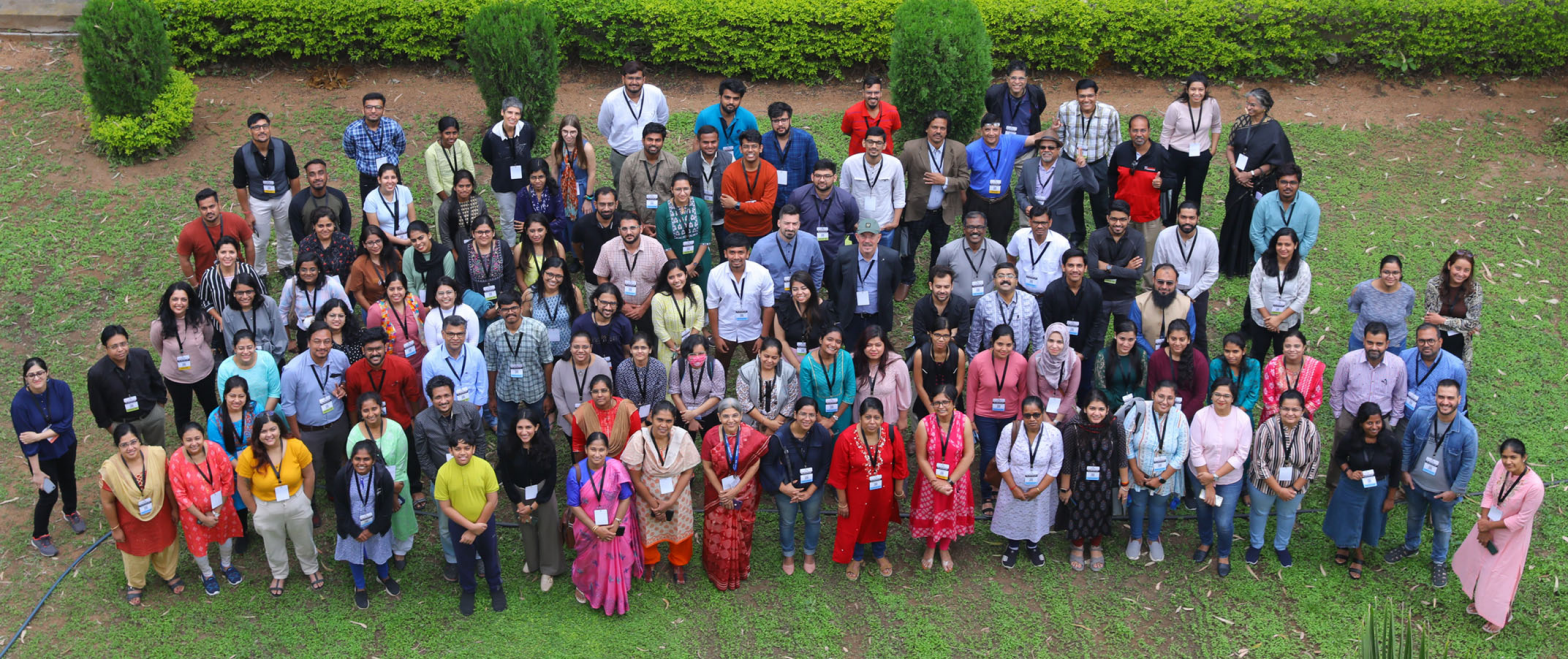
Participants of the India | EMBO lecture course held at Hyderabad, India from Oct 31-Nov 4, 2022.

**Fig. 2.**
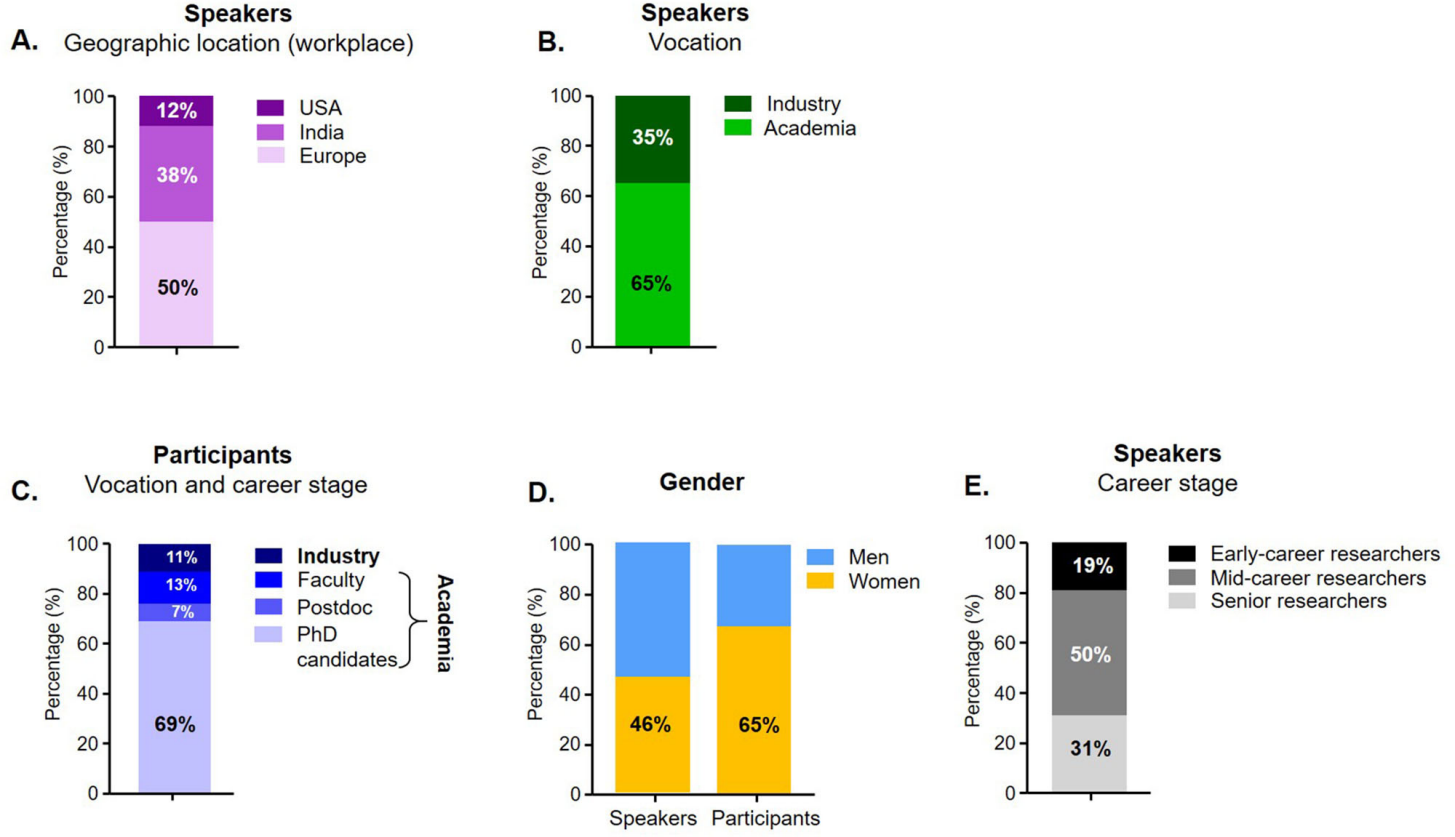
Speaker (*n*=26) and participant (*n*=99) distributions at the India | EMBO: MPS lecture course.

The meeting included 26 invited speakers working at laboratories across the globe (Figs 1 and 2A) on various aspects of organoids and OoC systems. The speakers hailed from both academic and industry backgrounds (Fig. 2B). It was attended by 99 participants of various career stages (Fig. 2C) and included industrialists, PhD candidates, postdocs and staff scientists from the US, Europe and India. Notably, the Indian participants hailed from ten states and two union territories across India. 26% of the participants are currently residing in tier 2/3 cities (less-developed) in India. In addition, 46% of the speakers and 65% of the participants self-identified as women (Fig. 2D). Lastly, 19% of the speakers (Fig. 2E) were early-career researchers (early career: within 10 years of receiving their PhD; mid-career: within 20 years of receiving their PhD; and senior researchers: experienced and established leaders in the field).

## Opening and keynote lecture

The opening lecture was delivered by the Secretary of Department of Biotechnology (DBT), Ministry of Science and Technology (Government of India) who spoke about various emerging technologies, including MPS that are transforming the future of therapeutics. The participation of a key official from DBT helped to highlight the role of MPS amongst government and regulatory bodies. A virtual keynote lecture was given by Dr Donald Ingber (Director of the Wyss Institute for Biologically Inspired Engineering at Harvard University, USA). His group has been a pioneer in developing microfluidic OoC or ‘living 3D cross-sections of human organs’. Their human lung airway-on-chip has been used to study cystic fibrosis and various respiratory diseases, such as influenza and COVID-19 (Huh et al., 2010).

## Understanding human development and disease using MPS models

There were four sessions on day 1 and 2 that were centred around understanding human development and disease using MPS models. Understanding the emergence of complex organs with different cellular fates from a uniform mass of cells is still one of the fundamental questions in biology.

The talks in this session were focussed on using iPSC-derived organoid models to understand brain and eye development, cell-fate regulation and disease pathophysiology (Mahato et al., 2022; Sriram et al., 2020; Susaimanickam et al., 2017). Mimicking human neurological disorders in rodent models is often challenging due to the species-specific differences in the complexity of the human brain. Thus, brain organoids present a unique window to study human-specific pathways during neurodevelopment and various neurological disorders (Eichmüller and Knoblich, 2022). Also, the iPSC-derived organoids can provide an unlimited supply of tissue-specific stem cell sources for developing novel regenerative therapies, particularly for organs such as the brain and retina, that show minimal repair ability in adult tissues. The second session had a specific focus on understanding neurological diseases using MPS models, including designing the cortical and mid-brain organoids This included iPSC-derived ‘brain spheres’, which contain all brain cell types, except microglia (Pamies et al., 2017) and was used to test neurodevelopment and developmental toxicity. Cerebral organoids consist of mostly cortical neurons and have a low abundance of midbrain dopaminergic neurons, which play a key role in Parkinson's disease (PD), making them unsuitable to model PD. Thus, it was discussed how genetic and high-content image analysis in midbrain organoids provides an opportunity to study PD.

The fundamental differences in cellular signalling pathways between model animals and humans also manifest in differential responses to pathogens and drug candidates. One such example is wound infection where differences in rodent and human wound healing mechanisms render them unsuitable as models (Grada et al., 2018). One of the talks described a ‘macrofluidic’ wound infection-on-chip device that also incorporated an *in vivo* wound milieu along with biofilms (integral components of chronic wounds) (Kadam et al., 2021). Two other talks were focussed on vessel-on-chip lined with human endothelium to model atherosclerosis (Jain et al., 2016) and a 3D-printed lung model to study pneumonia, asthma, tuberculosis, cystic fibrosis, and COVID-19 (Nandeesha et al., 2022 preprint).

Cancer organoids and tumour-on-chip systems can also be used to model the initiation and progression of cancer, and in a dedicated session, the talks focussed on mimicking various genetic and phenotypic characteristics of tumours. This included the multicellular architecture, tissue-tissue interface, chemical and mechanical properties of tumour spheroids (Nguyen et al., 2018). Tumour microenvironment, migration and its invasive properties remain one of key research areas in cancer biology. In this context, the effect of environmental stress and tumour-stromal crosstalk were also discussed (Gayan et al., 2022). Treatment protocol in cancer often calls for a combinatorial drug paradigm, and testing a combination of drugs using a microfluidic tumour-on-chip model was also discussed in this session.

## Design and challenges in MPS models

Various parameters need to be considered while designing a microfluidic OoC including microfabrication, compartmentalisation of tissue chambers, controlling transport processes, tailoring each chamber according to a specific tissue, biomechanical properties, creating a controlled and reproducible microenvironment, sourcing the relevant cells for building the system, etc. In this session, it was discussed how all these factors need to come together while designing an organ chip. Many challenging areas in the field, including generating multi-cell type tissues, combining organoid and OoC technologies, creating an interconnected multi-organ-on-chip, incorporating microbiome and immune cells into the microfluidic system and various microfabrication techniques were discussed (Goyal et al., 2022).

Organoids form through the process of self-organisation where a homogenous population of cells undergo symmetry breaking events to generate a patterned structure. Thus, to generate organoids with a high degree of reproducibility, it is essential to understand the principles of self-organisation, which was also one of the areas of discussion.

Cells and tissues inside our body do not exist in isolation and are exposed to different kinds of strains and mechanical stresses in the form of blood flow, extracellular matrix stiffness, and forces exerted by surrounding cells and tissues. Thus, incorporating these cues into a model system is also essential to mimic the human system accurately (Dahl-Jensen and Grapin-Botton, 2017). The talks in one of the sessions, therefore, focussed on mechanical properties of cells and tissues, and discussed how stress, toxicity, and efficacy can be measured and modulated in *in vitro* MPS systems using advanced imaging tools, phenotypic and genotypic signatures, quantitative measurements, and theoretical modelling. The physical cues from the underlying substrate were also elaborated as a critical parameter that influences cellular behaviour in tumour spheroids (Acharekar et al., 2023). One of the industry talks shared a disruptive technology developed as a workstation solution by combining MPS models composed of well-characterised progenitor cells and tissue systems with a trained digital platform. The resulting assay system was used to predict safety and efficacy concerns for humans (Dasari et al., 2022).

## Using MPS models in drug discovery

OoC are being increasingly leveraged by the pharmaceutical sector for drug development and safety assessments (Vulto and Joore, 2021). In one session dedicated to MPS in the pharmaceutical sector, the talks focussed on studying immune-related toxicity by detecting key events in advanced human cell systems. Drug-induced liver injury (DILI) remains one of the key reasons for drug withdrawal from the market attributing 30-45% to the attrition rate. Thus, scalable solutions for DILI testing, and strategies to validate these systems were discussed. In addition, use of spheroid models from cell lines and primary cells for drug discovery was also discussed.

## Setting standards and roadmap for MPS models

One important aspect of enabling a wider adoption of these emerging *in vitro* systems is to ensure their reproducibility, reliability and credibility across laboratories globally. In this session, various limitations and sources of variabilities of *in vitro* experiments and the salient features of Good Cell Culture Practices (GCCP) were discussed (Pamies et al., 2020, 2022). The internationally recognised OECD guidance document on Good *in vitro* Method Practice (GIVIMP) [OECD (2018)] is helping to support MPS method developers and end-users to establish these new, technologically complex *in vitro* methods in academic, industrial or government laboratories. It was highlighted that any *in vitro* MPS study should provide information about cells or tissues used (test system) to arrive at the measurements, the detection method, the method used for dose selection, control and reference chemicals used, specific experimental conditions, data analysis, acceptance criteria applied, validity of the data, reporting of results and uncertainties. This would improve the quality of submitted methods, accelerate their acceptability for biomedical, industrial, clinical and regulatory use, and ultimately reduce the experimental bias of derived *in vitro* data.

## Networking sessions

As many of our participants were students and early-career researchers, there was a dedicated networking session on career opportunities. It was an animated discussion that included questions on careers in academia, industry and opportunities. Some of the striking questions pertained to skills that one must build to be successful in science, the importance of a PhD over work experience, and transitioning from running your own project to managerial positions. Another session presented a networking opportunity on industry profiles, job trajectories and collaborations. This session had interesting questions on how students from state universities can raise their awareness of industry careers, how young founders of start-ups can support themselves in the initial stages, and how one can define partnerships and roles in a collaboration. There was a noticeable wave of emerging interest among Indian students in industry careers.

## Workshop

**Fig. 3.**
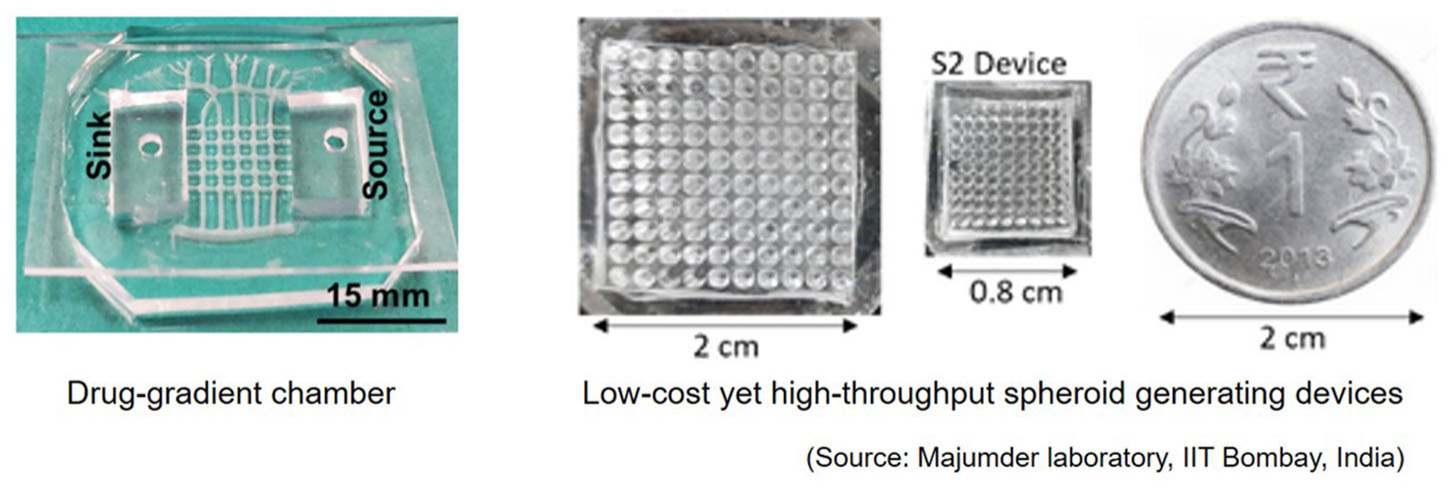
Microfabrication and setting-up of MPS models during the hands-on session.

We also had a three-day workshop on microfabrication for MPS models conducted by one of our speakers. Participants were involved in the preparation of OoC using polymers, setting up of drug-gradient devices and the generation and characterisation of human cancer cell spheroids (Fig. 3). These experiments helped the participants visualise the assembly of an OoC, providing a tangible call back to the MPS models they had spent the day learning about from the lecture course speakers.

## Impact

There is a great enthusiasm to understand and use MPS in India. Organisation of this meeting was crucial, as work in this area remains rudimentary in India, and the meeting equipped the participants with a vision for the future. The participation of government representatives in the meeting helped get more eyeballs on MPS as the next big frontier of research. Impetus has been created to prepare guidance documents for good cell and tissue culture practices in India. Entrepreneurs in this area who described their journey have served as role models for many others to venture forward with more confidence.

**Fig. 4.**
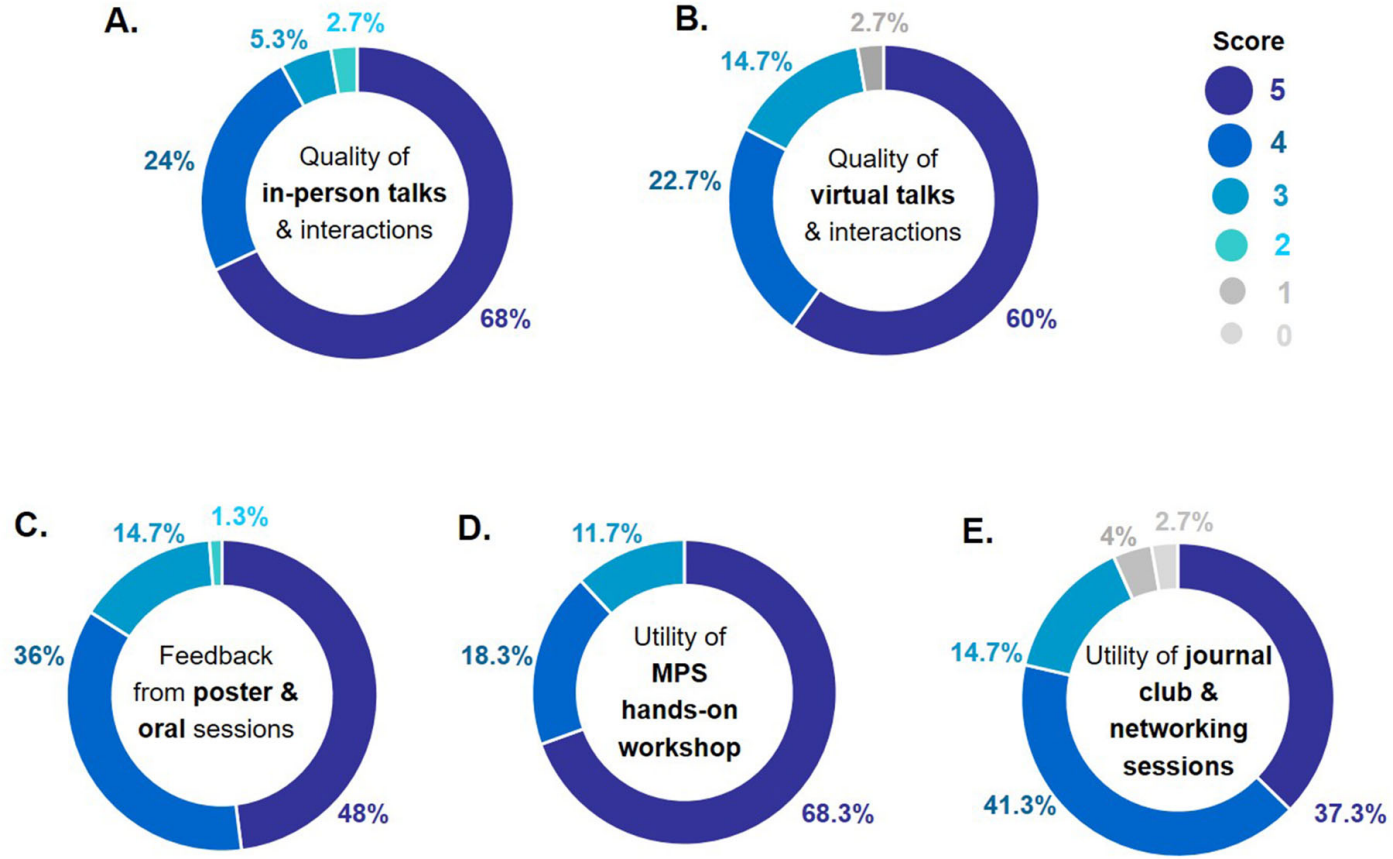
Feedback on the India | EMBO MPS lecture course from 75 attendees (68 participants and seven speakers).

The feedback for the India | EMBO MPS lecture course was overwhelmingly positive (Fig. 4). Of note, the virtual talks and speaker interactions were at par with in-person talks and did not present a hurdle in communication. The other activities, which included poster and oral presentations by early-career researchers, an MPS workshop, journal clubs and networking sessions, proved to be truely valuable additions.

## Conclusion and vision for the future

The India | EMBO lecture course was a great learning experience. Particularly, it was a joy to see the animated back-and-forth between the speakers and participants. For many participants, it was their first physical conference after COVID-19, made all the more valuable by the registration, accommodation and travel waivers. In addition, participants could avail themselves of childcare grants. This helped seven participants attend the meeting. In future meetings, CPHMS would like to bring more industry stakeholders to the table, to bring the perspective of end-users and the pharmaceutical sector in MPS research. In addition, based on the overwhelming response and need for hands-on experience, a dedicated two-day pre- or post-conference workshop for students has been planned.

Last year, CPHMS also conducted a roundtable meeting that included representatives from government bodies, regulatory bodies, academia and industry in an attempt to understand the needs of the MPS field in India (Parvatam, 2022). Several recommendations emerged from this meeting including the setting up of a dedicated Centre for Excellence (CoE) that brings together MPS researchers working in various domains, such as engineering, cell biology, optics, *in silico* tools, mathematical modelling, etc. This CoE could also work towards bringing together MPS expertise in different sectors, such as cosmetics, food, medical technology, etc., which could interface with and facilitate each other. In addition, there is a need to develop guidance documents for general cell and tissue culture practices in the Indian context, and to create awareness and harmonisation with the global MPS standards and protocols. These guidance documents and standards would help in ensuring intra- and inter-lab reproducibility, leading to increased confidence and greater adoption of these methods across different sectors. To advance these recommendations to actionable outcomes, CPHMS plans to form Translational Working Committees (including regulatory and government bodies, academia and industry professionals) that could meet on a regular basis to enable the translation of MPS technologies from bench to market.

Recently, the Union Health Ministry of India has introduced amendments to the New Drugs and Clinical Trials Rules, 2019, which validates the use of various non-animal methods, including MPS for drug safety and efficacy testing. If approved, India will become only the second country in the world (after the US) to adopt new technologies such as MPS, which can significantly reduce animal testing involved in novel drug approvals. In addition, DBT also recently released a dedicated Call for Proposal for organoid and stem cell-based models for disease and drug testing. It makes us believe that India has certainly put the right foot forward when it comes to pre-clinical research, and lecture courses such as these would help in providing forums for education, training, collaboration, and discussions in this emerging area.
